# General Medicine*Read at the Palestine Meeting East Texas Medico-Chirurgical Society, July 6, 1900.

**Published:** 1900-12

**Authors:** J. H. Joyce

**Affiliations:** Buffalo, Texas


For Texas Medical Journal.

### General Medicine.*

BY J. H. JOYCE, M. D., BUFFALO, TEXAS.

The subject given me by the committee on program is one of great dimensions, and as I understand it, could be treated of as medicine in general, as used by the human family in its varied forms and methods, but I presume that I should speak of it especially, as in the hands of the general practitioner, and in a more restricted sense should not take in even that large a field; as the general practitioner is considered among people of the country to be a man supposed to know all about medicine, surgery, gynecology, etc.

Medicine is defined as, "The science and art of preserving health, and preventing and curing disease," "the healing art"; including also the science of obstetrics. General medicine in this age of the world, in its application to disease, has not made the rapid strides that can be said of surgery. I mean that we do not understand thoroughly its action on all pathological conditions, hence, we often grope our way in the dark, feeling for some hidden something to combat and hold in check the ravages of disease. We are forced to stand in awe and watch the fearful onslaught of the tubercle bacilli on the human family! It is true that great advancement has been made in the way of letting the patient die easily and offering a respite for, in many instances, a number of years, yet more people are dying today from tuberculosis than from any other disease. On the other hand, there is a decrease in the frequency and in the mortality of typhoid fever all over the world, which decrease is probably due to improved sanitation and a better knowledge of the methods which should be employed in this malady. Allow me to say, that, I fear that medicine in the hands of the general practitioner has been and now is a greater factor for harm than for good. I say this advisedly; for instance, we will take some of the more

---

*Read at the Palestine Meeting East Texas Medico-Chirurgical Society, July 6, 1900.

powerful therapeutic agencies, such as nitro-glycerine—this potent remedy in the hands of a man who does not understand thoroughly its action on the heart, will often "shoot" his patient into that "land, from whose bourne no traveler has returned."

As medicine is the science of prevention, as well as the cure of disease, we will say that if the medical fraternity were worthy, and appreciated by the laity as it should be, many deaths could be averted, and long days and nights watching over the sick would almost be a thing of the past. (Let me remark right here, that to gain that confidence and to be ready at all times and under all circumstances to alleviate the sufferings of humanity involves one of the greatest questions that pertains to medicine—in the hands of the general practitioner.) The laity, from lack of confidence in the medical fraternity and oftentimes to save a doctor's bill, go to the drug store and do their own prescribing, and it is too often the case the doctor is called when no human agency could possibly do any good.

The fact that the progressive ones of the fraternity fail to get wholesome legislation on the practice of medicine, is due to the laggards in the fold, and to the prevailing sentiment among the people against the medical man. The laity have such a meager conception of how medicine should be controlled, that they take pride in having their representatives throttle all bills that come before the legislature, that have for their object the welfare of the people. I will be glad to see the day when the people will regard the legitimate study and practice of medicine as being the only means of prolonging life. The great amount of medicine in patented forms scattered over the country is an indication of the manner in which we are held by the people in general. If the use and practice of medicine could be kept within the control of the informed and up-to-date medical man, a new era would dawn upon the world—the pretender would hide his head, and medicine would ultimately reach its highest place in the prevention and cure of disease.

Therapeutics and pathology are so intimately connected with each other, that unless the latter be well understood theoretically, as well as practically, it is almost impossible to be successful therapeutists. It is true that occasionally empirical practice may succeed in effecting cures, but he alone who is well grounded in pathology can administer remedies with a hope of anything like uniform or permanent success. We should know the physiological actions of drugs on the system in its normal condition, so that we may know what results to expect in disease. If we are thoroughly useful and successful in our line of work, we should understand the *nature* of the disease we are called upon to correct. If we are thorough in physical diagnosis and understand the remedies at our command, we may hopefully enter into our field of labor. To this should be added a knowledge of their indications and contra-indications, as well as of their combinations which increase or diminish the medicinal activity of the various drugs—such an amount of knowledge is only to be attained by many years of study and experience and close observation, but every step which is made towards acquiring this information will render the practitioner so much the more efficient in the discharge of his professional duties.

When we understand more thoroughly the seat and nature of some obscure diseases, and when we understand more intelligently the *modus operandi* of medicine on the human frame, then we may be enabled, by attacking the cause of the disease, with proper remedies, to eliminate it at once from the system. We should be more definite in our application of medicine to disease; every symptom should be minutely examined, and oftentimes the absence of a symptom should be sufficient to constitute one. My observation teaches me that the great majority of general practitioners through the country, and in many instances in large towns, do not keep abreast of the times in the fundamental principles of medicine. The study of medicine is the most profound one in the universe, and the most astonishing thing to contemplate is the fact that so many half educated, as well as absolutely ignorant, men are following it as a profession. It is absolutely alarming to note the many pretenders who enter the field of general medicine. I am glad that the medical colleges are becoming more careful as to the applicant's literary attainments, and I verily believe none but college men should ever be allowed to matriculate. Then a term of four years *hard* application to medical study would prepare the student for some efficient work in the general practice.

It is true that there is much guess work in the application of medicine to disease. We often do not take time to look into the physical, moral, and social conditions of our patient as we should. The system, as we know, is often out of harmony with normal itself, simply from moral or social conditions. As practitioners we should study the general surroundings and environments of our patients so that we may be enabled to eliminate every obstruction to the proper action of medicine.

